# Unexpected uterine body placenta accreta spectrum with placenta previa in a subsequent pregnancy after uterine artery embolization: a case report

**DOI:** 10.1186/s12884-022-05031-0

**Published:** 2022-09-13

**Authors:** Shunya Sugai, Kaoru Yamawaki, Kazufumi Haino, Koji Nishijima

**Affiliations:** Department of Obstetrics and Gynecology, Niigata University Medical and Dental Hospital, 1-757 Asahimachi-dori, Chuo-ku, Niigata, 951-8510 Japan

**Keywords:** Hysterectomy, Placenta accreta, Placenta previa, Postpartum hemorrhage, Uterine artery embolization

## Abstract

**Background:**

A subsequent pregnancy after uterine artery embolization (UAE) raises several concerns, one of which is placenta accreta spectrum (PAS). Placenta previa is the strongest risk factor for PAS, which is most likely to occur in the lower uterine segment. PAS without placenta previa (i.e., uterine body PAS) is considered relatively rare.

**Case presentation:**

A 35-year-old woman, gravida 2 para 1, had undergone UAE for postpartum hemorrhage due to uterine atony after vaginal delivery in her previous pregnancy. She developed placenta previa during her subsequent pregnancy and was therefore evaluated for PAS in the lower uterine segment. On the basis of examination findings, we considered PAS to be unlikely. During cesarean section, we found that the placenta was not detached from the uterine body, and the patient was determined to have uterine body PAS. Ultimately, a hysterectomy was performed.

**Conclusions:**

PAS can occur in a subsequent pregnancy after UAE. When a subsequent pregnancy after UAE is accompanied by placenta previa, it is important to maintain a high index of suspicion of uterine body PAS without being misled by the presence of placenta previa.

## Background

Uterine artery embolization (UAE) is a treatment option for uncontrolled postpartum hemorrhage (PPH). The main advantage of UAE is the preservation of fertility. However, there is concern about the effect of UAE on a subsequent pregnancy. A systematic review revealed an increased risk of placenta accreta spectrum (PAS) in a subsequent pregnancy after UAE [1].

PAS is associated with considerable maternal morbidity because of PPH and, in the most severe cases, death. Prenatal diagnosis allows for multidisciplinary care and improved outcomes [2]. PAS occurs in 11.10% of patients with placenta previa [3]. In contrast, it reportedly occurs in only 0.18% of patients without placenta previa [4]. Placenta previa is considered the strongest risk factor for PAS. In other words, PAS tends to occur in the lower uterine segment, and the development of uterine body PAS is relatively rare [5]. The true prevalence of uterine body PAS is unknown, and it is unlikely to be diagnosed in the prenatal period [5, 6]. Diagnosis of PAS in the lower uterine segment with placenta previa using ultrasonography and magnetic resonance imaging (MRI) has been reported [2]. This condition tends to be expected in the prenatal period with a thorough evaluation [5, 7].

We herein report a case of uterine body PAS with placenta previa, which was unexpected in the prenatal period, in a subsequent pregnancy after UAE.

## Case presentation

**Fig. 1:**
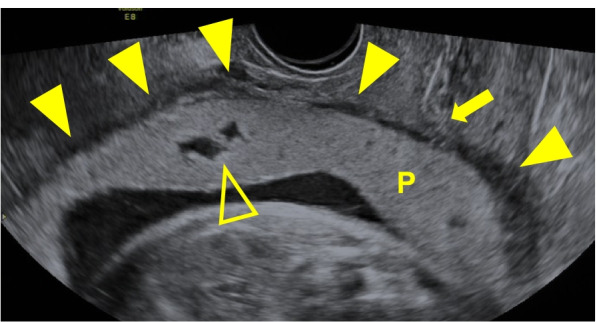
Transvaginal ultrasonography at 33 weeks’ gestation. A few small lacunae (hollow arrowhead) can be seen, but the retroplacental clear zone is preserved (solid arrowhead). The internal cervical os is indicated by an arrow. P: placenta

**Fig. 2:**
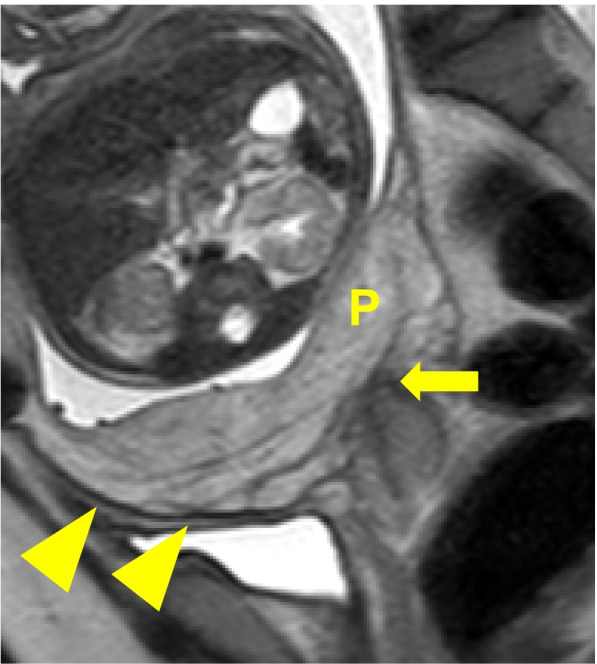
Sagittal T2-weighted magnetic resonance image at 33 weeks’ gestation. The thin hypointense interface between the placenta and the myometrium is preserved (arrowheads). The internal cervical os is indicated by an arrow. P: placenta

A 35-year-old woman, gravida 2 para 1, had undergone bilateral UAE with a gelatin sponge for PPH due to uterine atony after vaginal delivery in her previous pregnancy. The patient developed no UAE-related complications, and menstruation resumed. She subsequently conceived naturally and was found to have placenta previa. We performed a thorough examination with ultrasonography and MRI at 33 weeks’ gestation. The placenta was present on the anterior to lateral wall of the uterus, and the examination findings were consistent with total placenta previa. We found no evidence of PAS in the lower uterine segment (Figs. 1 and 2). We confirmed that her fetus was well-developed and in a state of well-being.

**Fig. 3:**
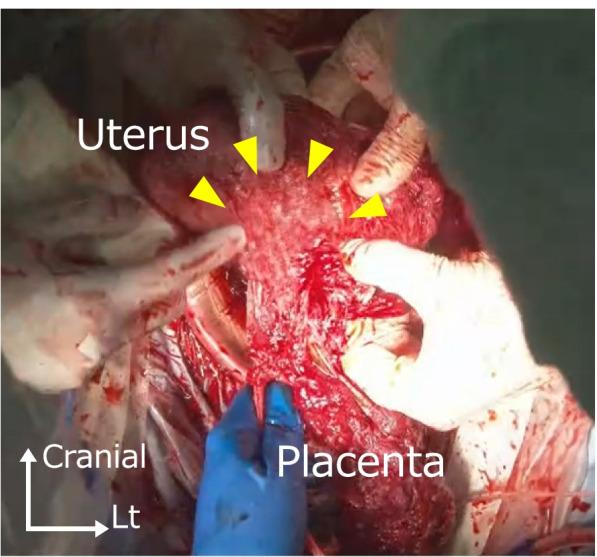
Intraoperative image. The uterus is inverted and the placenta is manually detached. The arrowheads indicate areas where the placenta is adherent

The patient underwent a cesarean section at 37 weeks’ gestation because of placenta previa. She delivered a healthy female neonate (birth weight of 2906 g, Apgar score of 8 at 1 min) by an oblique incision in the lower uterine body to avoid the placenta as much as possible. The placenta spontaneously detached from the lower uterine segment, but detachment of the placenta from the uterine body did not readily occur. We therefore manually detached the placenta (Fig. 3). We clinically diagnosed the patient with uterine body PAS with placenta previa. The uterus did not contract, and bleeding persisted. Therefore, an intrauterine balloon tamponade device with 100 mL of sterile liquid was inserted, and the uterus was sutured. The bleeding finally stopped, and the surgery was completed. The total blood loss was 2940 mL.

**Fig. 4:**
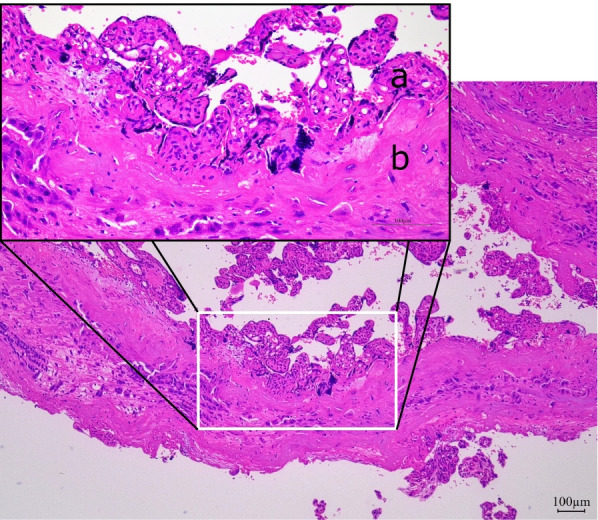
Pathologic examination of the manually dissected placenta. The chorionic villi are in direct contact with the myometrium (a: chorionic villi, b: myometrium). Hematoxylin and eosin, original magnification × 4 and × 20

After 1 h 30 min, 1040 mL of bleeding was observed from the drainage tube of the intrauterine balloon tamponade device. The patient’s systolic blood pressure was 119 mmHg, and her pulse rate was 76 beats/min. Her hemodynamics were stable. However, her uterus was not contracting, and active bleeding continued. She had no desire to preserve her fertility, and she had developed an allergic reaction to the contrast media at the time of her previous UAE. Therefore, we performed a subtotal hysterectomy instead of UAE. The surgery was uneventful. The total blood loss was 145 mL. The patient received 8 units of red blood cells and 6 units of fresh-frozen plasma throughout the procedure. Pathologically, she was diagnosed with placenta accreta because the chorionic villi were in direct contact with the myometrium (Fig. 4). Her postoperative condition was good, and she was discharged 7 days after surgery.

## Discussion and conclusion

PAS is more likely to occur in patients who have than have not undergone UAE. Even with the presence of placenta previa, evaluation of the lower uterine segment alone is not sufficient because of the possibility of uterine body PAS, as in the present case.

UAE is the treatment of choice for PPH refractory to pharmacological measures and intrauterine tamponade. The effectiveness of UAE is high, and a systematic review revealed a 90% success rate [8]. Additionally, in a previous study, UAE versus hysterectomy for refractory PPH significantly reduced blood loss, and there was no difference in the hemostasis rate [9]. The greatest advantage of UAE is probably its ability to preserve fertility. However, various obstetric complications have been reported in pregnancies subsequent to UAE. A systematic review revealed that a history of UAE for PPH was a risk factor for PAS in a subsequent pregnancy [1]. Interruption of uterine arterial blood flow by UAE causes damage to the endometrium. This is supported by post-UAE pathology findings showing necrotizing endometritis and myometritis [5]. Damage to the endometrium is thought to prevent normal decidualization, causing abnormally deep placental villi and trophoblastic invasion and thus leading to the development of PAS [10]. Other risk factors for PAS include placenta previa, prior cesarean section, intrauterine procedures, use of assisted reproductive technology, a history of PAS, a history of PPH, and twin gestation [11]. Other reports have indicated that smoking is a risk factor for PAS [12]. Furthermore, research has shown a unique association between endometriosis and PAS, although the cause is unknown [13]. Typically, PAS associated with placenta previa occurs in the lower uterine segment. In our case, the placenta previa and uterine body PAS were combined. We consider that the greatest risk factor for PAS in our case was a history of UAE. The relationship between a history of UAE and placenta previa is unclear [1].

In general, diagnosis of uterine body PAS is difficult, and there is no clear method to evaluate this condition. Therefore, clinicians must rely on the criteria for evaluating PAS in the lower uterine segment with placenta previa. It may be useful to evaluate uterine body PAS by ultrasonography in terms of loss of clear space, presence of lacunae, and thinning of the myometrium [5].

PAS occurs in the lower uterine segment in 880 of 100,000 women, whereas PAS in the uterine body is found in 5 of 100,000 women [5]. Therefore, the presence of placenta previa places women at high risk and prompts us to check for PAS in the lower uterine section. However, PAS without placenta previa (i.e., uterine body PAS) has a low index of suspicion. In pregnancies with the aforementioned risk factors, clinicians should have a high index of suspicion for PAS and perform a thorough examination regardless of the presence of placenta previa.

**Fig. 5:**
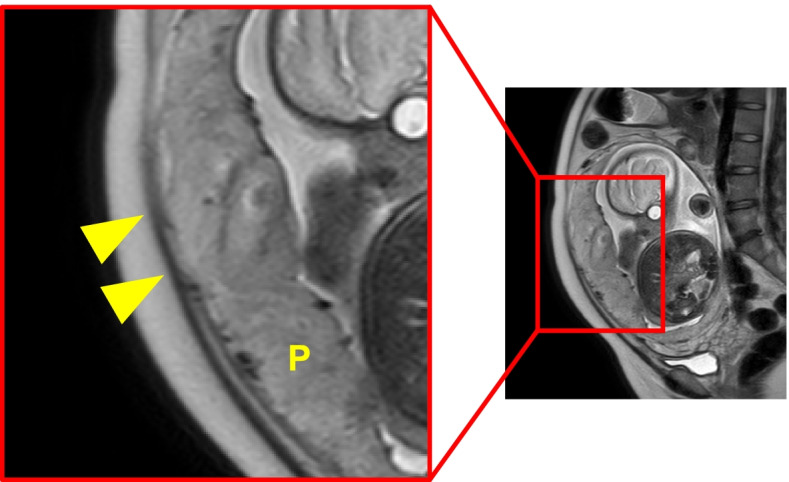
Sagittal T2-weighted magnetic resonance image at 33 weeks’ gestation. The thin hypointense interface between the uterine body placenta and the myometrium appears to have disappeared (arrowheads). P: placenta

Our patient had undergone UAE for PPH in a previous pregnancy. After we manually detached the placenta during cesarean section in the present pregnancy, hysterectomy was performed ultimately because of uterine atony. Because of the presence of placenta previa, we performed detailed ultrasonography and MRI for PAS in the lower uterine segment. However, we did not specifically evaluate the patient for uterine body PAS. Looking back on this case, we retrospectively observed that the thin hypointense interface between the uterine body placenta and the myometrium on MRI had disappeared (Fig. 5). Although not definitive, this finding (an “interface sign”) may be an indicator of PAS [14].

In conclusion, patients who have undergone UAE have a risk of PAS in a subsequent pregnancy. In a post-UAE pregnancy with placenta previa, it is natural to perform an evaluation to determine the presence or absence of PAS in the lower uterine segment. We emphasize that evaluation of the patient for possible uterine body PAS is also essential without being misled by the presence of placenta previa.

## Data Availability

All data related to this report are available from the corresponding author on reasonable request.

## Abbreviations

MRI: Magnetic resonance imaging
PAS: Placenta accreta spectrum
PPH: Postpartum hemorrhage
UAE: Uterine artery embolization

## References

[1] Matsuzaki S, Lee M, Nagase Y, Jitsumori M, Matsuzaki S, Maeda M (2021). A systematic review and meta-analysis of obstetric and maternal outcomes after prior uterine artery embolization. Sci Rep.

[2] Silver RM, Branch DW (2018). Placenta Accreta Spectrum. N Engl J Med.

[3] Jauniaux E, Grønbeck L, Bunce C, Langhoff-Roos J, Collins SL (2019). Epidemiology of placenta previa accreta: a systematic review and meta-analysis. BMJ Open.

[4] Matsuzaki S, Mandelbaum RS, Sangara RN, McCarthy LE, Vestal NL, Klar M (2021). Trends, characteristics, and outcomes of placenta accreta spectrum: a national study in the United States. Am J Obstet Gynecol.

[5] Badr DA, Al Hassan J, Salem Wehbe G, Ramadan MK (2020). Uterine body placenta accreta spectrum: A detailed literature review. Placenta.

[6] Carusi DA, Fox KA, Lyell DJ, Perlman NC, Aalipour S, Einerson BD (2020). Placenta Accreta Spectrum Without Placenta Previa. Obstet Gynecol.

[7] Erfani H, Fox KA, Clark SL, Rac M, Rocky Hui SK, Rezaei A, et al. Maternal outcomes in unexpected placenta accreta spectrum disorders: single-center experience with a multidisciplinary team. Am J Obstet Gynecol. 2019;221(4):337.e1-.e5. 10.1016/j.ajog.2019.05.035 PMC8651298 31173748

[8] Zhang XQ, Chen XT, Zhang YT, Mai CX (2021). The Emergent Pelvic Artery Embolization in the Management of Postpartum Hemorrhage: A Systematic Review and Meta-analysis. Obstet Gynecol Surv.

[9] Liu Z, Wang Y, Yan J, Li J, Liu X, Zhang L (2020). Uterine artery embolization versus hysterectomy in the treatment of refractory postpartum hemorrhage: a systematic review and meta-analysis. J Matern Fetal Neonatal Med.

[10] Jauniaux E, Collins S, Burton GJ (2018). Placenta accreta spectrum: pathophysiology and evidence-based anatomy for prenatal ultrasound imaging. Am J Obstet Gynecol.

[11] Conturie CL, Lyell DJ (2022). Prenatal diagnosis of placenta accreta spectrum. Curr Opin Obstet Gynecol.

[12] Kyozuka H, Yamaguchi A, Suzuki D, Fujimori K, Hosoya M, Yasumura S (2019). Risk factors for placenta accreta spectrum: findings from the Japan environment and Children's study. BMC Pregnancy Childbirth.

[13] Matsuzaki S, Ueda Y, Nagase Y, Matsuzaki S, Kakuda M, Kakuda S (2022). Placenta accreta spectrum disorder complicated with endometriosis: systematic review and meta-analysis. Biomedicines..

[14] Kapoor H, Hanaoka M, Dawkins A, Khurana A (2021). Review of MRI imaging for placenta accreta spectrum: Pathophysiologic insights, imaging signs, and recent developments. Placenta.

